# Beyond a biased binary: A perspective on the misconceptions, challenges, and implications of studying females in avian behavioral endocrinology

**DOI:** 10.3389/fphys.2022.970603

**Published:** 2022-09-21

**Authors:** Kristina O. Smiley, Sara E. Lipshutz, Abigail A. Kimmitt, M. Susan DeVries, Kristal E. Cain, Elizabeth M. George, Kristen. M. Covino

**Affiliations:** ^1^ Centre for Neuroendocrinology and Department of Anatomy, School of Biomedical Sciences, University of Otago, Dunedin, New Zealand; ^2^ Center for Neuroendocrine Studies and Department of Psychological and Brain Sciences, University of Massachusetts-Amherst, Amherst, MA, United States; ^3^ Biology Department, Loyola University Chicago, Chicago, IL, United States; ^4^ Department of Ecology and Evolutionary Biology, University of Michigan, Ann Arbor, MI, United States; ^5^ Department of Biological Sciences, University of Wisconsin-Whitewater, Whitewater, WI, United States; ^6^ School of Biological Sciences, University of Auckland, Auckland, New Zealand; ^7^ Biology Department, Texas A&M University, College Station, TX, United States; ^8^ Biology Department, Loyola Marymount University, Los Angeles, CA, United States

**Keywords:** testosterone, progesterone, prolactin, challenge hypothesis, sex hormones, female birds, sex differences, estradiol

## Abstract

For decades, avian endocrinology has been informed by male perspectives and male-focused research, leaving significant gaps in our understanding of female birds. Male birds have been favored as research subjects because their reproductive behaviors are considered more conspicuous and their reproductive physiology is presumably less complex than female birds. However, female birds should not be ignored, as female reproductive behavior and physiology are essential for the propagation of all avian species. Endocrine research in female birds has made much progress in the last 20 years, but a substantial disparity in knowledge between male and female endocrinology persists. In this perspective piece, we provide examples of why ornithology has neglected female endocrinology, and we propose considerations for field and laboratory techniques to facilitate future studies. We highlight recent advances that showcase the importance of female avian endocrinology, and we challenge historic applications of an oversimplified, male-biased lens. We further provide examples of species for which avian behavior differs from the stereotypically described behaviors of male and female birds, warning investigators of the pitfalls in approaching endocrinology with a binary bias. We hope this piece will inspire investigators to engage in more comprehensive studies with female birds, to close the knowledge gap between the sexes, and to look beyond the binary when drawing conclusions about what is ‘male’ versus ‘female’ biology.

## Introduction

From Berthold’s first experiment in the 19th century documenting that the testes are necessary for the expression of male sex characteristics in chickens (Berthold and Quiring, 1944), the field of avian endocrinology has been dominated by male-focused research. Although females have been well-studied in some domesticated species such as poultry, the study of natural biological variation in other female birds has received considerably less attention in the fields of behavioral endocrinology and evolutionary biology (Hrdy, 1986; Cotton et al., 2006; Shansky, 2019; Shansky and Murphy, 2021), particularly in studies examining mate quality and reproductive strategies. This sex bias in the literature was demonstrated in a systematic review, which found that 84% of avian physiology, ecology, and reproduction studies between 2003 and 2011 involved male birds, whereas only 58% involved female birds (Caro, 2012). A 10-years follow up study found that this discrepancy persists at similar rates in the current literature (Kimmitt, 2020). In addition to being understudied, female variation in key life history traits is typically examined in the context of how such variation affects males, rather than an interesting and important subject itself (Alonzo and Warner, 2000; Rosenthal and Ryan, 2022). Such male-biased perspectives have misled research on female birds for decades, causing major misconceptions in the field of behavioral endocrinology.

**FIGURE 1:**
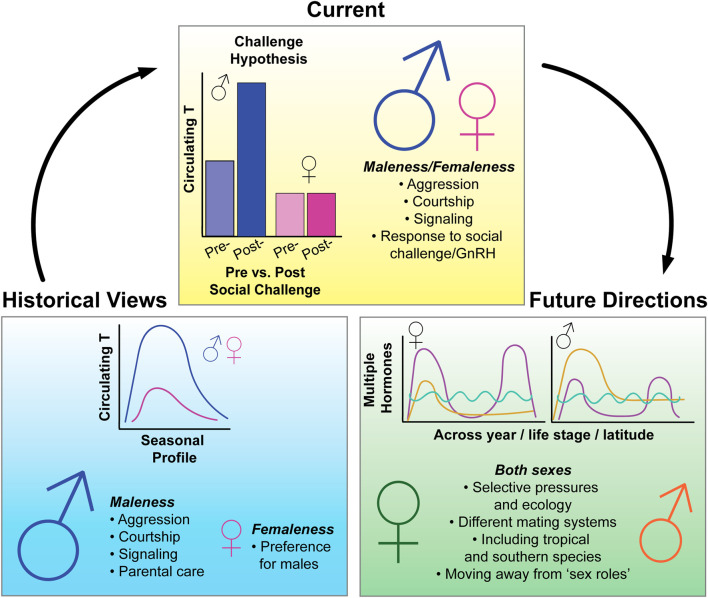
Summary figure of historical, current, and future state of research in avian behavioral neuroendocrinology. Historically, the field of avian endocrinology has focused on seasonal variation of testosterone (T) and its effects on “male traits”, with little emphasis on how hormones may be affecting similar traits in females. Recent research, which has taken a more female-directed approach, has overturned some previously held dogmas of the field, such as the Challenge Hypothesis (Wingfield 1990)—which seeks to explain variation in T levels across the breeding season. The inclusion of more female-perspectives in avian research has also demonstrated that similar hormones can have similar effects in both females and males and previously thought “male traits” such as dominance, aggression, and bright coloration, also persist in females. In the future, we hope to see more emphasis on female-research, leading to more even study of the sexes on a range of hormone-mediated traits and behaviors. However, to develop more inclusive research programs, we must put increased emphasis on studying and understanding the unique selective pressures and ecology of both sexes, conduct research that focuses on the spectrum of variation for all individuals, and become aware of potential sources of sex bias in our research methodology and design. Note that figures schematics are for illustrative purposes.

Although female avian endocrinology has received increased attention in the past 20 years, male-dominated research programs and male-biased study designs persist (Figure 1). In this perspective piece, we address inherent barriers with current research methods that lead to these biases. In the second part of this piece, we highlight recent advances that showcase the importance of studying female avian endocrinology and challenge historic applications of an oversimplified, male-biased lens. Lastly, we offer solutions to address and overcome male bias in future research and increase female inclusivity. Our perspective shares important insights with many recent publications on the inclusion of female animals in biology (Shansky, 2019; Orr et al., 2020; Rosvall et al., 2020) including birds (Ball and Ketterson, 2008; Caro, 2012; Kimmitt, 2020). This is by no means an exhaustive list of topics for which significant improvements can be made. Rather, we consider this a starting point for investigators interested in designing female-inclusive research.

## Barriers to our understanding of avian endocrinology in females

Here, we highlight specific examples of logistical, technical, and cultural biases that have impeded our understanding of hormones in female birds.

### Capture techniques

One commonly used explanation for persistent sex bias in avian endocrinology is that males are more conspicuous or aggressive than females, and therefore easier to capture and study in the field (Caro, 2012; Kimmitt, 2020). However, as evidenced by recent research efforts studying free-living females (e.g., Cain and Ketterson, 2012; DeVries et al., 2015; Covino et al., 2018; George and Rosvall, 2018; Kimmitt et al., 2019; Lipshutz et al., 2021), a better understanding of the natural history of female birds can make sampling less difficult. For example, in cavity-nesting species, females can be more easily captured in artificial nest boxes compared to traditional mist netting techniques (George and Rosvall, 2018; Bentz et al., 2021). In open cup nesting species, females can be easier to capture prior to egg laying (Kimmitt et al., 2019; Needham et al., 2019), as they might be more active or aggressive in the early breeding season (Cristol and Johnsen, 1994; Sandell and Smith, 1997; Reichard et al., 2018). Females in open cup nesting species can also be captured in peak breeding season if researchers devote time to find nests and use the appropriate sex as a lure, as females are often more aggressive towards other females (Cain and Ketterson, 2012; DeVries and Jawor, 2013; Graham et al., 2017). Overall, females are not necessarily “more difficult” to capture, but their capture likelihood may be limited when using methods developed for studying males.

### Seasonal bias

Research efforts in avian biology are biased toward reproductive seasons (Marra et al., 2015), when capture likelihood may be most likely to differ by sex. During non-breeding seasons, including migration, females and males may be equally likely to be captured, making this a prime opportunity to ask questions about both sexes (e.g., Covino et al., 2018; Covino et al., 2015; Covino et al., 2017; DeVries et al., 2011). Distinguishing between females and males in sexually amorphic species can be difficult when breeding anatomical features (e.g., brood patch, cloacal protuberance) are absent, but this can be resolved with inexpensive molecular sexing techniques (Fridolfsson and Ellegren, 1999). Monomorphic species also provide a natural control for sex-related biases in sampling and/or data (e.g., behavioral assays).

### Tropical vs. temperate bias

Most studies in behavioral endocrinology focus on temperate-breeding migrants, with the implied assumption that these species are models for birds more generally. However, the vast majority of avian species are year-round tropical residents (Hau et al., 2008). Drastic differences in environmental and life-history traits between temperate and tropical species likely have considerable effects on hormone-behavior relationships for both sexes (Levin and Wingfield, 1992; Hau et al., 2004). Despite the dearth of research on tropical species, the available evidence undermines traditional hypotheses, especially as related to the role of testosterone (hereafter, “T”) in aggression and song (Peters et al., 2001; Moore et al., 2004; York et al., 2016). For example, in tropical and southern hemisphere species, females defend territories (Levin, 1996; Hall, 2004; Tobias et al., 2011; Cain and Langmore, 2016), sing in the context of territorial defense (Illes, 2015; Riebel et al., 2019; Loo and Cain, 2021), and are brightly colored (Dale et al., 2015; Price, 2015), all traits which are more traditionally associated with T and male-male competition in the northern hemisphere (Hau and Goymann, 2015).

### Captive vs. wild studies

Captive experiments, where environmental conditions can be manipulated, are important for testing hypotheses in avian endocrinology. However, females of many species do not readily enter reproductive conditions in captivity, whereas males do (Rosvall et al., 2020). Females of domesticated species like canaries (*Serinus canaria*) and zebra finches (*Taeniopygia guttata*) readily breed in captivity and are models for endocrinology (e.g., Adkins-Regan, 1999; Williams and Martyniuk, 2000; Hurley et al., 2008; Smiley and Adkins-Regan, 2016; Madison et al., 2020), but domesticated species may not best represent how physiological traits evolve in the wild. Some research groups have successfully studied reproduction in captive females by hand-rearing chicks (Baptista and Petrinovich, 1986) or providing spacious aviaries (Caro et al., 2007; Perfito et al., 2015; Lindner et al., 2021). Both solutions are time-consuming and expensive, however, which may limit sample size and feasibility. Expanding the number of species for which females can be studied in captivity requires funding sources to support these efforts and costs.

### Measuring hormones

The ability to measure hormones from blood in free-living birds gave birth to the subdiscipline of “field endocrinology” (Wingfield et al., 2020). However, there are several logistical barriers to quantifying hormone concentrations in female birds. For instance, female T concentrations may be lower than the detection limits of enzyme linked immunosorbent assays (ELISA) designed to measure higher “male-typical” T levels. Additional hormones can be difficult to measure in both sexes, due to limitations of current ELISA kit sensitivity and sample volumes (e.g., estradiol) or a lack of reliable, commercially available ELISA kits that work for birds (e.g., prolactin, IGF1, LH, and FSH). Liquid Chromatography/Mass Spectrometry (LC/MS) is a promising method for quantifying multiple hormone concentrations from small volumes of blood or tissue (Jalabert et al., 2021; Munley et al., 2022). However, LC/MS has drawbacks, as it requires expensive equipment and rigorous calibration. Alternative methods of non-invasive sampling (e.g., fecal or feather) could also increase sample sizes for measuring hormone variation in both sexes (Chávez-Zichinelli et al., 2010; Chávez-Zichinelli et al., 2014). Although these sampling methods are not equivalent to plasma sampling, they reveal integrated hormone profiles on longer timescales (Bortolotti et al., 2008), which may be beneficial depending on the research question. We are optimistic that further advancements will support the quantification of hormone levels in female birds.

### “Sex roles”—flipped and reversed

Substantial emphasis has been placed on conventional “sex roles” for female and male animals, in line with the Darwin-Bateman paradigm of sexual selection (Janicke et al., 2016; Gonzalez-Voyer et al., 2022). This binary framing reinforces what is expected for sex-specific courtship, competition, parental care, and their endocrinological correlates, thereby limiting how we examine and interpret variation in natural history (Ah-King and Ahnesjö, 2013). We also highlight the problematic framing of birds as “sex-role reversed”, in which females compete for multiple male mates (i.e., social polyandry), and males conduct the majority of parental care (Emlen and Oring, 1977). In these avian systems, the degree of sexual dimorphism in average T secretion varies, depending on whether males are conducting parental care or seeking courtship (Lipshutz and Rosvall, 2020). Similarly, in cooperatively or group breeding species, sex differences in hormones are often considerably less than differences between breeders and non-breeders, though female levels are rarely reported (Pikus et al., 2018). We argue that sex (i.e., the default framing of “sexual dimorphism”) is not necessarily the main predictor of variation in avian endocrinology. We envision a future framework for which phenotypic traits like behavior, morphology, and hormone levels can be viewed across a spectrum, rather than categorically by sex.

### Misleading terminology

Many misconceptions about the endocrinology of females stem from misleading terminology. The language we use can create and reinforce bias, which constrains our understanding of hormones and behaviors. For example, the pervasive labeling of T and its metabolites as “male hormones'' with “masculinizing effects,” and estrogen and progesterone as “female hormones'' with “feminizing effects” (e.g., 68, a widely used undergraduate textbook) presents a false binary that these hormones only have sex-specific functions, when in reality these hormones are functionally important in both sexes. Whereas titers of T may be higher in males than in females, T’s functional capabilities are similar in both sexes (Staub and De Beer, 1997). Furthermore, hormonal values exist along a continuous spectrum, and a binary approach that emphasizes differences between groups might ignore important similarities and within-group variation (Williams, 2008). In some cases, hormonal variation between the sexes can be seen as overlapping bell curves (Muck and Goymann, 2011; Goymann and Wingfield, 2014), and variation within sex categories is greater than variation between them. Mislabeling hormones as the “male hormone” or “maternal hormone” disregards their broader regulatory functions and can lead us to overlook their importance in all individuals.

## Advances that highlight the importance of studying female avian endocrinology

Below, we highlight some specific examples of how studying hormones in female birds has broadened our understanding of avian endocrinology, by facilitating the testing of old and new hypotheses.

### Testosterone is more than a “male hormone”

Our understanding of T’s role in female behavior is in its infancy (Rosenthal and Ryan, 2022), despite decades of study on T in male birds (Wingfield et al., 1990; Goymann et al., 2019). Recent work reveals T-behavior relationships, which have been classically described as “male”, also exist in females. For example, research in female songbirds has demonstrated that both T and aggression are elevated early in the breeding season (Cain and Ketterson, 2012; George and Rosvall, 2018), and that territorial aggression positively correlates with circulating T levels (Lipshutz and Rosvall, 2021). Meanwhile, many purported effects of T on classically “male” behavior are instead mediated by the “female” hormone estradiol. For example, in certain brain regions T is converted to estradiol, and both hormones activate either the androgen or estrogen receptor to promote singing behavior (Frankl-Vilches and Gahr, 2018) and aggression (Ubuka and Tsutsui, 2014).

Even as researchers began to recognize the biological relevance of T in females, it has nevertheless been studied within a male-typical framework. For example, the Challenge Hypothesis established an important framework for answering questions concerning T’s role in modulating male social behavior (Wingfield et al., 1990). Yet, as males of more species were assessed, conflicting results emerged (Wingfield et al., 2019) and recent modification suggests that the presence of females, rather than male competitors, explains variation in male T levels within the breeding season (Goymann et al., 2019). In female birds, assessment of the Challenge Hypothesis suggests that T elevations do not accompany acute social challenges in most species examined thus far (Rosenthal and Ryan, 2022). However, T’s influence on aggressive behavior cannot entirely be ruled out (Cain and Ketterson, 2012; Lipshutz and Rosvall, 2021; George et al., 2022). To build a conceptual framework that works for T-behavior relationships in females, future work should account for the unique selective pressures relevant to their life history.

### Prolactin is more than a “maternal hormone”

Whereas androcentric terminology has led our field to overlook important aspects of female biology, the same can be said for using gynocentric terminology and male biology. One such example is the anterior pituitary hormone prolactin, which is most well-known for its regulation of mammalian lactation and maternal behavior. There is little research focused on prolactin in males—a rare female bias in research! However, biparental care is widespread in birds, with both males and females participating in egg incubation and/or chick provisioning in over 80% of avian species (Cockburn, 2006). In some species, including ring doves and pigeons, both females and males produce and regurgitate “crop milk,” a nutrient-rich substance secreted from the crop sac organ to feed young (Lehrman, 1955; Buntin et al., 1991). Though most other birds do not produce crop milk, in virtually all avian species studied to date, prolactin levels increase just before hatching in all individuals (both sexes) that provide parental care (Smiley, 2019). In both male and female zebra finches, the rise in prolactin before hatching is required for parenting behaviors (Smiley and Adkins-Regan, 2018). Inter- and intra-specific differences in paternal investment have also been linked with prolactin levels in male songbirds (Van Roo et al., 2003; Badyaev and Duckworth, 2005). Together, these experiments demonstrate that prolactin plays a similar role in both female and male birds, and is far from solely a “maternal hormone.”

### Females are active participants in courtship behaviors

Female courtship behavior is often interpreted from a “male perspective” or is neglected altogether. For instance, breeding territory quality and resource access has been well-studied in males. In contrast, much less attention is given to which females mate with these males, how females acquire high-quality resources, and whether females choose mates based on male traits or territory characteristics (Hasegawa et al., 2012; Cain and Rosvall, 2014). In avian endocrinology, studies of courtship often center on T’s role in regulating elaborate male traits (Riters et al., 2011), while little is known about the endocrine regulation of mating signal perception in females. Although courtship is generally thought to be male-driven, hormones such as progesterone and gonadotropin-releasing hormone stimulate copulation solicitation and other courtship behaviors, which are an active female mating signal (Maney et al., 1997; Smiley et al., 2012). Given that courtship is a critical component of both male and female reproductive success, we encourage studies that highlight the bidirectionality of behavioral and physiological mechanisms.

### Female birds sing

Bird song is a classic subject in behavioral endocrinology, and the link between T and singing is well-established in males. The bulk of song research has focused on a highly derived clade of northern hemisphere migrants that are seasonally territorial (Riebel et al., 2019; Rose et al., 2022), but songbirds evolved in the southern hemisphere, where birds often sing year-round (Rose et al., 2022; Theuerkauf et al., 2022). Thus, patterns in this group may not be generalizable (Gahr, 2014; Ball, 2016). Further, our understanding of hormones and song is primarily based on males, or to a lesser extent experimentally manipulated females in species without female song (Riebel et al., 2019; Rose et al., 2022; Catchpole, Slater, Song). However, female song is widespread and ancestral (Ball, 2016), often functioning in an analogous manner to male song—acquiring and defending critical reproductive resources (Langmore, 1998; Cain et al., 2015; Hall et al., 2015; Odom et al., 2014). Neuroanatomical comparisons of sex differences in the song control system have found that HVC (used as a proper name) and RA (robust nucleus of the arcopallium) volumes are larger in males, even in species for which females sing similarly, or more often (Ball, Balthazart). However, androgen receptor distribution appears similar in the song control nuclei of females for which both sexes sing (Gahr, 2014). Much remains to be studied on the role of hormones in regulating female song development, neuroendocrine processes, and performance (Riebel et al., 2019; Rose et al., 2022; Rouse, 2022).

## Where do we go from here?

Here, we propose and reflect on next steps for a more inclusive field of avian endocrinology.

### Males are not the baseline

Research on males has been retrofitted to females instead of coming from first principles. We argue that by grounding research in the natural history of female birds, we can make better predictions about hormones-behavior relationships. This requires countering the biased ideas that females do not compete, are always maternal, and are relatively interchangeable (i.e., do not exhibit functionally important variation). In particular, more work is needed to identify the specific selection pressures that shape female behavior and physiology (Buchanan and Fanson, 2014). We encourage studies that evaluate whether a hormone has the same function in both males and females, or whether it has sex-specific effects, and under which developmental, physiological, social, and ecological circumstances we expect to find these similarities and differences. Changing our perspective and assumptions alters the questions we ask and how we test them, and helps us avoid the errors that are too often engrained in experimental design. This will facilitate progress on developing a deeper and more integrative understanding of how phylogeny, ecology, and physiology interact to shape female behavior, and by extension, population persistence and dynamics. We offer a set of questions to ask next time a research paper is being critically evaluated or better yet, while a study is being designed, to increase awareness of potential sex biases (dBox 1).

DBOX 1 **Beyond Male-Centered Research**

The **Bechdel test** is a tool for examining representation of women in entertainment, asking whether women-identifying characters are represented and whether they talk to each other about anything other than a man-identifying character (roughly half of movies fail the test). We argue a similar test should be applied studies of avian endocrinology and beyond, as females and other sex/gender minorities are largely understudied. Next time you read a manuscript, ask yourself the following questions:

1) Do the authors discuss the potential effects of sex (or gender in human-centered research) on variation in the trait of interest?

2) Do the authors report sample sizes of each sex and are the sexes equally represented?

2a) If the research is centered on one sex, do the authors discuss previous findings in the other sex (es)?

3) Do the authors include sex as a fixed effect in their statistical models?

Bonus: Does the research challenge pre-existing sex-related biases?

### More and different data

A collective goal in biology is to establish patterns and determine the mechanisms driving those patterns. Reviews and meta-analyses are key for evaluating the predictive strength of these hypotheses. However, such work requires empirical data on a broad array of taxa, locations, and life-histories. With each new study on a different aspect of the behavioral endocrinology, we have found new patterns or nuances to previously well-supported patterns, and we are still lacking in general theories for many aspects of female avian endocrinology. This is particularly evident as we have moved away from the original model species—namely migratory sparrows, zebra finches, and canaries - and towards species with different life-histories and ecologies, such as tropical, non-migratory, cooperative breeders, polyandrous females, etc. As research expands to other species in other parts of the world, these gaps are slowly filling in.

### Measuring the complete system of signal and reception

Studying circulating hormones alone cannot provide a complete understanding of a bird’s underlying physiology, as this approach ignores other crucial components of endocrine signaling systems, such as receptors, enzymes, and carrier proteins (Hau, 2007; Ketterson et al., 2009). Focusing on only circulating hormones can lead to a binary understanding of these endocrine systems. As an example, we might conclude that sex differences in circulating levels of T have some functional importance across species, but this singular focus on T signal ignores the many other components of the androgen signaling system, including tissue sensitivity to the signal, and the rate of conversion to other hormones (Rosvall, 2013; Lipshutz et al., 2019; Schuppe et al., 2020). Therefore, progress in our understanding of the hormonal phenotypic continuum must include a more comprehensive study of these endocrine axes, in both females and males.

### Revising our language and perpetuating correct terminology

One challenge to building a more inclusive avian endocrinology is that many biased ideas are heavily entrenched in the minds of the general, well-educated public. Outdated concepts are perpetuated by inaccurate textbooks (Raven, 2020) and popular media which use misleading headlines to generate clicks, when the reality is much less sensational. We encourage researchers to avoid terms like “masculinization” and instead use terms like “androgenization” or “increased T”, to help us move away from the false binary of “male” versus “female” hormones. We hope this shift in terminology will also help address misinterpretations and misuses of our research by the public, fellow scientists, and legislators alike.

### Increasing diversity in scientists

We all bring biases to our research, but through self-awareness of positionality, greater inclusion of diverse backgrounds, and explicit reckoning with barriers and biases, we can minimize these blind spots (Kamath et al., 2022). As the number of scientists of underrepresented genders increases in our community, so do the number of studies that include different perspectives, make different assumptions, and examine questions from a new angle (Baran, 2018; Haines et al., 2020; Tang-Martínez, 2020). Increasing diversity among scientists is an important antidote to the issues we have addressed here.

dBox 1: To combat potential sex-bias research, we propose a set of questions to ask next time a research paper is being critically evaluated. These same questions can be applied when designing new studies. Discussing these concepts with trainees and other colleagues can increase awareness and can encourage scientists to consider the ‘female-perspective’ in future research efforts.

## Data Availability

The original contributions presented in the study are included in the article/Supplementary Material, further inquiries can be directed to the corresponding author.
